# Adherence to Self-Monitoring of Blood Glucose and Its Related Factors Among Type 2 Diabetic Patients Attending Al-Ahsa Primary Health Care Centers in Saudi Arabia

**DOI:** 10.7759/cureus.65545

**Published:** 2024-07-27

**Authors:** Afnan Y AlRasheed, Hajer Hashim, Hassan Alrofaie

**Affiliations:** 1 Family Medicine, Ministry of Health, Al-Hofuf, SAU; 2 Obstetrics and Gynecology, King Faisal University, Al-Hofuf, SAU

**Keywords:** primary healthcare, saudi arabia, type 2 diabetes mellitus, adherence, self-monitoring of blood glucose

## Abstract

Background: Self-monitoring of blood glucose (SMBG) is a crucial component of diabetes management, but adherence remains suboptimal. This study aimed to evaluate adherence to SMBG among type 2 diabetic patients in Al-Ahsa, Saudi Arabia.

Methods: A cross-sectional study was conducted among 398 type 2 diabetic patients attending primary healthcare centers. Data were collected through face-to-face or virtual interviews and electronic health records. Adherence levels were categorized as low, moderate, and high.

Results: The majority of participants exhibited moderate adherence to SMBG (58.5%), while 27.1% had low adherence, and 14.3% were highly adherent. The use of oral hypoglycemic medications and insulin injections was associated with higher adherence (p<0.001). Comorbidities, physical exercise, diet, frequency of medical visits, and attendance at diabetes education sessions did not significantly influence adherence.

Conclusions: Suboptimal adherence to SMBG was observed among type 2 diabetic patients in Al-Ahsa. Targeted interventions addressing individual barriers and integrating technology may improve SMBG adherence and diabetes management.

## Introduction

Diabetes mellitus (DM) is a chronic metabolic disorder characterized by hyperglycemia resulting from defects in insulin secretion, insulin action, or both. Globally, the prevalence of DM, particularly type 2 diabetes mellitus (T2DM), has been increasing at an alarming rate [1]. According to the International Diabetes Federation (IDF), an estimated 537 million adults were living with diabetes in 2021, with the majority being diagnosed with T2DM [2]. This rise in prevalence is a significant public health concern due to the associated complications, healthcare costs, and the overall burden on healthcare systems.

Saudi Arabia is among the countries with the highest prevalence of diabetes. Recent statistics indicate that approximately 18.3% of the Saudi population, equivalent to 6.5 million people, are affected by diabetes, positioning Saudi Arabia as the second highest in the Middle East and the seventh globally in terms of diabetes prevalence [3,4]. The high prevalence of diabetes in Saudi Arabia can be attributed to several factors, including rapid urbanization, dietary changes, physical inactivity, and genetic predisposition [5]. Uncontrolled diabetes can lead to severe acute and chronic complications, which significantly impair the quality of life and increase morbidity and mortality rates [6]. Microvascular complications, such as diabetic retinopathy, nephropathy, and neuropathy, and macrovascular complications, including coronary artery disease, stroke, and peripheral vascular disease, are common among individuals with diabetes [7]. These complications not only affect the health and well-being of individuals but also impose a substantial economic burden on healthcare systems. In Saudi Arabia, the economic cost of diabetes was estimated at $2.4 billion in 2013, with projections reaching $6.5 billion by 2020 [8].

Effective management of diabetes focuses on maintaining blood glucose levels within a target range to prevent or delay the onset of complications [9]. Glycemic control is a critical component of diabetes management, typically assessed using the HbA1c test, which reflects average blood glucose levels over the past two to three months [10]. The American Diabetes Association (ADA) recommends an HbA1c target of less than 7.0% for most adults with diabetes, with individualized targets based on patient-specific factors [11]. Self-monitoring of blood glucose (SMBG) is a cornerstone of diabetes management, especially for patients on insulin therapy. SMBG involves the regular checking of blood glucose levels by the patient using a portable glucometer [12]. This practice provides immediate feedback on blood glucose levels, enabling patients to make informed decisions about their diet, physical activity, and medication [13]. Additionally, SMBG helps healthcare providers assess the effectiveness of treatment plans and make necessary adjustments to achieve optimal glycemic control.

Despite the benefits of SMBG, adherence to this practice remains a challenge for many patients. Several factors influence adherence to SMBG, including demographic characteristics, psychological barriers, and the perceived burden of self-monitoring [14]. Studies have shown that older age, lower educational levels, and lower income are associated with poorer adherence to SMBG. Psychological factors such as fear of testing, pain associated with finger pricks, and frustration with "poor" blood glucose readings also contribute to low adherence [14].

In Saudi Arabia, several studies have examined adherence to SMBG among patients with diabetes. A study conducted in the Eastern region of Saudi Arabia between 2011 and 2012 evaluated the impact of intensified SMBG combined with patient education programs on glycemic control [15,16]. The results indicated that patients who received intensified SMBG and education had significantly lower fasting blood glucose and HbA1c levels compared to those who did not receive the intervention [12]. This study underscores the importance of education and support in improving adherence to SMBG and achieving better glycemic control.

Despite the known benefits of SMBG, various challenges hinder patients from adhering to this practice. In a qualitative study, Gucciardi et al. identified several barriers to SMBG, including distressing emotions and thoughts, fingertip pain, and frustration about high blood glucose readings [17]. Similarly, a study by Nagelkerk et al. highlighted barriers such as lack of awareness of hypoglycemia and hyperglycemia symptoms, lack of social support, and difficulty in interpreting SMBG results [18]. These findings suggest that both emotional and practical factors need to be addressed to improve SMBG adherence.

DM, particularly type 2 diabetes, poses a significant public health challenge in Saudi Arabia due to its high prevalence and associated complications [4]. Effective management of diabetes through glycemic control is essential to prevent complications and improve patient outcomes [19]. SMBG is a vital tool in diabetes management, but adherence to this practice is often suboptimal [20]. By investigating adherence to SMBG and identifying related factors, this study aims to contribute to the development of targeted interventions to improve diabetes management in Saudi Arabia [21].

## Materials and methods

Study design

This study employs a cross-sectional design to evaluate adherence to SMBG among type 2 diabetic patients attending primary health care centers in the Al-Ahsa governorate, Saudi Arabia. The study was conducted over a one-year period, from April 2023 to April 2024.

Study area and settings

The research was carried out in multiple primary healthcare centers affiliated with the Ministry of Health in the Al-Ahsa governorate. Al-Ahsa is located in the Eastern Province of Saudi Arabia, and it is one of the largest oases in the world. The healthcare centers involved in this study are part of a well-established network providing comprehensive primary care services, including diabetes management.

Study population

The study population comprised adults over 18 years old diagnosed with T2DM who were virtually contacted by the selected primary healthcare centers. These patients were required to self-monitor their blood glucose levels using a glucometer.

Inclusion criteria

Participants were included in the study if they met the following criteria: diagnosed with T2DM, aged 18 years or older, using a glucometer for self-monitoring blood glucose, and provided informed consent to participate in the study.

Exclusion criteria

The following patients were excluded from the study: those with type 1 DM, patients with gestational diabetes, type 2 diabetes patients using continuous glucose monitoring systems, and patients under 18 years old.

Sample size and sampling technique

A simple random sampling technique was used to select participants from the patient records available at the primary healthcare centers. The target sample size was calculated based on the prevalence of type 2 diabetes in the Al-Ahsa governorate and the expected adherence rate to SMBG. A minimum sample size of 400 participants was determined to be adequate for the study.

Data collection

Data Sources

Data were collected from the Electronic Health Record (EHR) database of the primary healthcare centers and through face-to-face or virtual interviews. The EHR provided demographic and clinical information, while the interviews collected data on SMBG practices and attitudes.

Data Collection Tools

An interviewer-administered questionnaire was used as the primary data collection tool. The questionnaire was developed based on existing validated instruments and tailored to the study objectives. It consisted of three main sections.

Socio-Demographic Characteristics

The study collected information on age, sex, nationality, marital status, level of education, employment status, monthly income, and body mass index (BMI).

Diabetes Management

Participants provided details on the duration of diabetes, family history of diabetes, current diabetes treatment regimen (diet and exercise, oral hypoglycemic agents, insulin injections, or a combination), frequency of medical consultations for diabetes, and attendance at diabetes education sessions.

SMBG

The study also assessed the frequency and timing of SMBG and the level of glycemic control as indicated by the latest HbA1c measurement.

Data collection procedure

The data collection process was conducted in several phases.

Preparatory Phase

The preparatory phase involved obtaining ethical approval from the relevant authorities and the local ethics committee, training data collectors on the study protocol and the use of the data collection tools, and piloting the questionnaire on a small sample of patients to ensure clarity and appropriateness.

Fieldwork Phase

The fieldwork phase involved extracting patient records from the EHR database to identify eligible participants, contacting these patients to invite them to participate in the study and obtain informed consent, conducting face-to-face or virtual interviews using the structured questionnaire, and recording responses while cross-checking data for accuracy and completeness.

Data management and analysis

Data Entry and Cleaning

Data collected from the questionnaires were entered into a database using Microsoft Excel. The data were then cleaned to remove any inconsistencies, errors, or missing values. Two independent researchers cross-verified the data entry process to ensure accuracy.

Statistical Analysis

Data analysis was performed using IBM SPSS Statistics for Windows, Version 28 (Released 2021; IBM Corp., Armonk, New York, United States). Descriptive statistics were calculated to summarize the demographic characteristics, diabetes management practices, and SMBG adherence levels of the study participants. Continuous variables were presented as means and standard deviations, while categorical variables were presented as frequencies and percentages.

Inferential Statistics

To identify factors associated with SMBG adherence, inferential statistics were employed. The Pearson chi-square test was used to assess associations between categorical variables and SMBG adherence levels. For continuous variables, independent t-tests were used as appropriate. A p-value of less than 0.05 was considered statistically significant.

Ethical considerations

Ethical approval for the study was obtained from the Institutional Review Board (IRB) of the Ministry of Health. Written or verbal informed consent was obtained from all participants before their inclusion in the study. The confidentiality and privacy of participants were strictly maintained throughout the study. Data were anonymized and stored securely, with access restricted to the research team.

## Results

Table 1 presents a comprehensive overview of the demographic characteristics of the study participants. The majority of the participants were male (54%), and a significant portion of the participants were married (83%). Educational attainment varied, with the largest groups being those with below high school education (32%) and high school education (23%). In terms of employment status, most participants were either unemployed (34%) or employed (35%), with a smaller proportion being housewives (30%) and students (1%). The majority of the participants reported a monthly income of ≤6000 SAR (62%), reflecting a predominantly low-income population. Smoking prevalence was low, with only 11% having smoked a cigarette in the past week. In terms of BMI, a significant number of participants were classified as obese (58%), while 30% were overweight, and only 12% were of normal weight. Age distribution showed that most participants were in the 40-59 age group (62%), followed by the 60-79 age group (24%). The mean age of the participants was 52 years with a standard deviation of 10.90 years, indicating a middle-aged population with a wide age range.

**Table 1:** Demographics of study participants

Demographics	n	%
Sex		
Male	214	54.0
Female	184	46.0
Marital status		
Single	17	4.0
Married	329	83.0
Divorced	9	2.0
Widowed	43	11.0
Level of education		
Uneducated	60	15.0
Below High School	129	32.0
High School	90	23.0
Diploma	32	8.0
Bachelor	84	21.0
Postgraduate	3	1.0
Employment status		
Housewife	119	30.0
Unemployed	135	34.0
Employed	140	35.0
Student	4	1.0
Monthly income (SAR)		
≤6000	248	62.0
6001-10000	89	22.0
10001-20000	56	14.0
>20000	5	1.0
Smoked a cigarette in the past week		
Yes	43	11.0
No	355	89.0
BMI		
Underweight	0	0.0
Normal	49	12.0
Overweight	120	30.0
Obese	229	58.0
Age groups		
20-39	54	14.0
40-59	246	62.0
60-79	96	24.0
80-99	2	1.0
Mean age (years)	52	10.90

Table 2 presents the adherence levels to SMBG among type 2 diabetic patients. The majority of patients (233, 58.5%) were moderately adhered to SMBG, while 108 (27.1%) showed low adherence, and only 57 (14.3%) demonstrated high adherence. These results indicate that a significant proportion of patients are not fully compliant with the recommended SMBG practices, highlighting the need for targeted interventions to improve adherence rates and, consequently, glycemic control in this population.

**Table 2:** Adherence to self-monitoring of blood glucose (SMBG) among type 2 diabetic patients

Adherence level	n	%
Low adhered	108	27.1
Moderately adhered	233	58.5
High adhered	57	14.3
Total	398	100.0

Table 3 presents the distribution of adherence levels (low, moderate, high) to SMBG among type 2 diabetes patients, categorized by their HbA1c levels. The adherence is broken down into three categories of HbA1c: intensive, moderate, and poor glycemic control. The majority of patients (233, 59%) exhibit moderate adherence to SMBG, while only a small proportion (57, 14%) show high adherence. Notably, the highest adherence levels are observed in patients with moderate HbA1c, indicating that patients with better glycemic control are more likely to adhere to SMBG practices. However, the p-value of 0.155 suggests that the differences in adherence levels across the HbA1c categories are not statistically significant.

**Table 3:** Distribution of adherence to self-monitoring of blood glucose by HbA1c levels

HbA1c	Adherence	Total	Chi-square (χ^2^)	p-value
Intensive	Low adhered	32	129	3.75
	Moderately adhered	85		
	High adhered	12		
Moderate	Low adhered	38	132	
	Moderately adhered	75		
	High adhered	19		
Poor	Low adhered	38	137	
	Moderately adhered	73		
	High adhered	26		
Total	Low adhered	108	398	
	Moderately adhered	233		
	High adhered	57		

Table 4 presents the demographic characteristics of the study participants and their adherence to SMBG. The p-values indicate the statistical significance of the relationships between these characteristics and SMBG adherence. None of the variables showed a statistically significant association with adherence levels, suggesting that factors such as sex, marital status, education level, employment status, monthly income, smoking status, BMI, and age do not significantly influence adherence to SMBG in this sample. For sex, the adherence levels are similar between males and females. Among males, 57 (53%) were low adherents, 129 (55%) were moderately adherent, and 28 (49%) were highly adherent. Similarly, among females, 51 (47%) were low adherents, 104 (45%) were moderately adherent, and 29 (51%) were highly adherent, with a chi-square (χ^2^) value of 0.78 and a p-value of 0.678 indicating no significant difference.

**Table 4:** Demographic characteristics and adherence to self-monitoring of blood glucose (SMBG)

Demographics	Low adhered	Moderately adhered	High adhered	Total	Chi-square (χ^2^)	p-value
Sex						
Male	57 (53%)	129 (55%)	28 (49%)	214	0.78	0.678
Female	51 (47%)	104 (45%)	29 (51%)	184		
Marital status						
Single	5 (5%)	11 (5%)	1 (2%)	17	1.84	0.607
Married	93 (86%)	191 (82%)	45 (79%)	329		
Divorced	1 (1%)	6 (3%)	2 (4%)	9		
Widowed	9 (8%)	25 (11%)	9 (16%)	43		
Level of education						
Uneducated	12 (11%)	34 (15%)	14 (25%)	60	5.42	0.144
Below High School	30 (28%)	77 (33%)	22 (39%)	129		
High School	27 (25%)	53 (23%)	10 (18%)	90		
Diploma	10 (9%)	21 (9%)	1 (2%)	32		
Bachelor	29 (27%)	45 (19%)	10 (18%)	84		
Postgraduate	0 (0%)	3 (1%)	0 (0%)	3		
Employment status						
Housewife	27 (25%)	73 (31%)	19 (33%)	119	1.70	0.637
Unemployed	36 (33%)	77 (33%)	22 (39%)	135		
Employed	44 (41%)	81 (35%)	15 (26%)	140		
Student	1 (1%)	2 (1%)	1 (2%)	4		
Monthly income (SAR)						
≤ 6000	59 (55%)	150 (64%)	39 (68%)	248	7.34	0.06
6001-10000	29 (27%)	45 (19%)	15 (26%)	89		
10001-20000	20 (19%)	33 (14%)	3 (5%)	56		
> 20000	0 (0%)	5 (2%)	0 (0%)	5		
Smoked a cigarette in the past week						
Yes	10 (9%)	29 (12%)	4 (7%)	43	1.77	0.413
No	98 (91%)	204 (88%)	53 (93%)	355		
BMI						
Underweight	0 (0%)	0 (0%)	0 (0%)	0	3.07	0.380
Normal	8 (7%)	34 (15%)	7 (12%)	49		
Overweight	32 (30%)	72 (31%)	16 (28%)	120		
Obese	68 (63%)	127 (55%)	34 (60%)	229		
Age groups						
20-39	16 (15%)	33 (14%)	5 (9%)	54	3.88	0.420
40-59	69 (64%)	139 (60%)	38 (67%)	246		
60-79	22 (20%)	61 (26%)	13 (23%)	96		
80-99	1 (1%)	0 (0%)	1 (2%)	2		

Marital status also showed no significant association with SMBG adherence (χ^2^ = 1.84, p = 0.607). The majority of the participants were married, with 93 (86%) low adherents, 191 (82%) moderately adherent, and 45 (79%) highly adherent participants falling into this category. Single, divorced, and widowed individuals were distributed similarly across adherence levels. Education level did not significantly affect adherence to SMBG (χ^2^ = 5.42, p = 0.144). Participants with below high school education made up 30 (28%) low adherents, 77 (33%) moderately adherent, and 22 (39%) highly adherent individuals. Interestingly, uneducated participants constituted 12 (11%) low adherents, 34 (15%) moderately adherent, and 14 (25%) highly adherent participants. Employment status showed a non-significant association with adherence (χ^2^ = 1.70, p = 0.637). Unemployed participants were the most common among low adherents (36, 33%) and highly adherent (22, 39%) groups. Employed participants made up 44 (41%) low adherents but only 15 (26%) high adherents.

Monthly income was another factor without significant association (χ^2^ = 7.34, p = 0.06). A majority of participants earning ≤6000 SAR were low adherents (59, 55%), moderately adherent (150, 64%), and highly adherent (39, 68%). Higher-income levels were less represented among high adherents, with only 3 (5%) participants earning 10001-20000 SAR and none earning >20000 SAR being highly adherent. Smoking status in the past seven days did not significantly affect SMBG adherence (χ^2^ = 1.77, p = 0.413). Among those who had smoked, 10 (9%) were low adherents, 29 (12%) were moderately adherent, and 4 (7%) were highly adherent. Non-smokers constituted 98 (91%) low adherents, 204 (88%) moderately adherent, and 53 (93%) highly adherent participants.

Table 5 presents the relationship between various diabetes management factors and adherence levels to SMBG among type 2 diabetic patients, categorized as low, moderate, and high adherence. The data reveals several key findings. Patients with a family history of diabetes generally exhibited higher adherence levels to SMBG, although this association was not statistically significant (χ^2^ = 5.91, p = 0.052). The use of oral hypoglycemic tablets showed a significant association with higher adherence levels (χ^2^ = 28.90, p < 0.001), indicating that patients on these medications are more diligent in monitoring their blood glucose. Similarly, insulin injections were significantly associated with higher adherence (χ^2^ = 51.18, p < 0.001), suggesting that patients using insulin are more committed to SMBG. In contrast, no significant associations were found between adherence levels and physical exercise (χ^2^ = 1.34, p = 0.511) or diet (χ^2^ = 2.05, p = 0.358), implying that these lifestyle factors did not significantly influence SMBG adherence. The frequency of visits to a general practitioner (GP) also did not show a significant association with adherence levels (χ^2^ = 2.00, p = 0.765), indicating that regular consultations alone do not necessarily enhance SMBG adherence. Last, attending diabetes education sessions in the past seven days did not significantly impact adherence levels (χ^2^ = 2.35, p = 0.310), although those who attended the sessions exhibited slightly higher adherence.

**Table 5:** Relationship between diabetes management factors and adherence to self-monitoring of blood glucose (SMBG) GP: general practitioner

Factor	Low adhered	Moderately adhered	High adhered	Total	Chi-square (χ^2^)	p-value
Family history of diabetes						
Yes	99 (92%)	198 (85%)	54 (95%)	351	5.91	0.052
No	9 (8%)	35 (15%)	3 (5%)	47		
Oral hypoglycemic tablets						
No	17 (16%)	14 (6%)	0 (0%)	31	28.90	<0.001
Yes	91 (84%)	219 (94%)	57 (100%)	367		
Insulin injections						
No	89 (82%)	150 (64%)	3 (5%)	242	51.18	<0.001
Yes	19 (18%)	83 (36%)	54 (95%)	156		
Physical exercise						
No	60 (56%)	114 (49%)	30 (53%)	204	1.34	0.511
Yes	48 (44%)	119 (51%)	27 (47%)	194		
Diet						
No	62 (57%)	119 (51%)	34 (60%)	215	2.05	0.358
Yes	46 (43%)	114 (49%)	23 (40%)	183		
Frequency of GP visits						
Every three months	73 (68%)	161 (69%)	44 (77%)	278	2.00	0.765
Every six months	11 (10%)	13 (6%)	3 (5%)	27		
Monthly	21 (19%)	49 (21%)	8 (14%)	78		
Weekly	1 (1%)	3 (1%)	0 (0%)	4		
Yearly	0 (0%)	3 (1%)	1 (2%)	4		
Never	2 (2%)	4 (2%)	1 (2%)	7		
Diabetes education sessions in past seven days						
No	95 (88%)	197 (85%)	45 (79%)	337	2.35	0.310
Yes	13 (12%)	36 (15%)	12 (21%)	61		

## Discussion

The present study aimed to evaluate adherence to SMBG among patients with T2DM attending primary healthcare centers in the Al-Ahsa governorate of Saudi Arabia. The findings revealed that the majority of participants (58.5%) exhibited moderate adherence to SMBG, while 27.1% demonstrated low adherence, and only 14.3% were highly adherent. These results are consistent with previous studies conducted in Saudi Arabia and other countries, which have reported suboptimal adherence to SMBG among patients with diabetes [22-24].

The suboptimal adherence to SMBG observed in this study is concerning, as SMBG is a crucial component of effective diabetes management. Regular SMBG levels enable patients to make informed decisions about their diet, physical activity, and medication adjustments, ultimately leading to better glycemic control and a reduced risk of complications [25,26]. A study highlighted the positive impact of SMBG on glycemic control, showing that individuals who performed SMBG more frequently had lower HbA1c levels compared to those who monitored less frequently [27].

Interestingly, the present study found no significant association between demographic factors, such as sex, marital status, education level, employment status, monthly income, smoking status, BMI, and age, and adherence to SMBG [28,29]. These findings contradict previous studies that have reported associations between demographic characteristics and SMBG adherence [30]. For instance, a study found that older age, lower educational levels, and lower income were associated with poorer adherence to SMBG [31]. The lack of significant associations in the current study may be attributed to the homogeneous nature of the study population or the influence of other factors that were not captured in the study [32].

Regarding diabetes management factors, the present study revealed significant associations between the use of oral hypoglycemic medications and insulin injections and higher adherence to SMBG. Patients taking these medications were more likely to be moderately or highly adherent to SMBG. This finding is consistent with previous research, which has shown that patients on pharmacological treatments for diabetes tend to be more diligent in monitoring their blood glucose levels [33]. It is possible that patients on medication perceive a greater need to monitor their blood glucose levels to assess the effectiveness of their treatment and make necessary adjustments [34].

Interestingly, the presence of comorbidities, such as hypertension, high cholesterol, and obesity, did not significantly influence adherence to SMBG in the current study. This finding contradicts previous studies that have reported an association between comorbidities and SMBG adherence [35]. It is possible that the presence of comorbidities alone may not be a strong predictor of SMBG adherence, and other factors, such as perceived severity, disease knowledge, and self-efficacy, may play a more significant role [36].

The lack of significant associations between some diabetes management factors (e.g., physical exercise, diet, frequency of GP visits, and attendance at diabetes education sessions) and SMBG adherence in the current study is noteworthy. These findings suggest that simply providing educational interventions or encouraging lifestyle modifications may not be sufficient to improve SMBG adherence. A more comprehensive approach that addresses individual barriers, motivations, and self-efficacy may be necessary to enhance adherence to SMBG [37].

The findings of this study have important implications for healthcare professionals and policymakers in Saudi Arabia. The suboptimal adherence to SMBG highlights the need for targeted interventions to improve diabetes management and prevent complications. Healthcare providers should focus on identifying and addressing individual barriers to SMBG adherence, such as psychological factors, fingertip pain, and frustration with high blood glucose readings [38]. Providing tailored education, emotional support, and practical strategies can help overcome these barriers and empower patients to take an active role in their diabetes management.

Limitations

This study has several limitations that should be considered when interpreting the findings. First, the cross-sectional design of the study limits the ability to establish causal relationships between the variables examined. While associations between factors such as medication use and SMBG adherence were observed, the cross-sectional nature of the data does not allow for determining the direction of these relationships.

Second, the study was conducted in a specific geographic region, the Al-Ahsa governorate of Saudi Arabia. While this area has a large population and represents an important region for diabetes management, the findings may not be fully generalizable to the entire Saudi population or other regions with different demographic and cultural characteristics.

Future studies could address these limitations by employing longitudinal or prospective study designs, expanding the geographic scope to include multiple regions or a nationally representative sample, and using more objective measures of SMBG adherence, such as electronic monitoring devices or direct observation by healthcare providers.

Implications

The findings of this study have several important implications for healthcare practice, policy, and future research related to diabetes management in Saudi Arabia.

From a healthcare practice perspective, the suboptimal adherence to SMBG observed in this study highlights the need for targeted interventions to support and improve adherence among patients with type 2 diabetes. Healthcare providers should prioritize identifying and addressing individual barriers to SMBG adherence, such as psychological factors, pain associated with finger pricks, and frustration with high blood glucose readings. Tailored education, emotional support, and practical strategies can help overcome these barriers and empower patients to take an active role in their diabetes self-management.

Furthermore, the integration of technology into diabetes care presents promising opportunities to enhance SMBG adherence and overall disease management. Mobile applications, continuous glucose monitoring systems, and telemedicine solutions have shown the potential to improve diabetes outcomes by providing real-time feedback, reminders, and support. Implementing and promoting the use of such technologies in Saudi Arabia's healthcare system could facilitate better monitoring, enhance patient engagement, and ultimately improve glycemic control and patient outcomes.

From a policy perspective, healthcare policies and programs should prioritize the promotion of SMBG adherence and diabetes self-management education. Collaborative efforts between healthcare providers, policymakers, patient advocacy groups, and technology companies can help develop effective strategies to address the unique challenges faced by patients in Saudi Arabia. Such efforts may include increasing awareness, providing access to affordable monitoring devices and supplies, and fostering a supportive environment for diabetes self-management.

Additionally, further research is needed to explore the complex interplay of factors influencing SMBG adherence, including psychological, social, cultural, and environmental factors. Qualitative studies can provide deeper insights into the barriers and facilitators experienced by patients, while longitudinal studies can examine the long-term effects of interventions on adherence and clinical outcomes.

## Conclusions

In conclusion, the present study revealed suboptimal adherence to SMBG among patients with T2DM attending primary healthcare centers in the Al-Ahsa governorate of Saudi Arabia. While demographic factors did not significantly influence adherence levels, the use of oral hypoglycemic medications and insulin injections was associated with higher adherence to SMBG. These findings underscore the need for targeted interventions to improve SMBG adherence and overall diabetes management in this population. Addressing individual barriers, providing tailored education and support, and integrating technology-based solutions may be effective strategies to enhance adherence and empower patients to take an active role in their care. Moreover, healthcare policies and programs should prioritize the promotion of SMBG adherence and diabetes self-management education. Collaborative efforts between healthcare providers, policymakers, and patient advocacy groups are crucial to developing effective strategies that address the unique challenges faced by patients in Saudi Arabia.
